# Design of Tunable Broadband Graphene-Based Metasurface with Amplitude-Phase Modulation

**DOI:** 10.3390/ma16134633

**Published:** 2023-06-27

**Authors:** Huixia Jiang, Lili Sheng, Yumei Luo, Liang Meng, Weiping Cao

**Affiliations:** 1 Guangxi Key Laboratory of Wireless Wideband Communication and Signal Processing, School of Information and Communication, Guilin University of Electronic Technology, Guilin 541004, China; 2 School of Electronic Information and Automation, Guilin University of Aerospace Technology, Guilin 541004, China; 3 Guangxi Key Laboratory of Information Materials, Guilin University of Electronic Technology, Guilin 541004, China; 4 The 34th Research Institute of China Electronics Technology Group Corporation, Guilin 541004, China

**Keywords:** graphene, metasurface, reflection amplitude phase, code

## Abstract

Due to the growing scarcity of spectrum resources in the low-frequency band, the requirement of beam-reconfigurable antennas in the millimeter wave band is urgent. In this paper, a W-band graphene-based metasurface working in a broad bandwidth is proposed with reflective amplitude coding. Here, graphene sheets play a dual role in radiating and regulating electromagnetic waves. By adjusting the Fermi levels of graphene, the reflective amplitude and phase of the metasurface can be modulated simultaneously, enabling multi-beam switching and beam deflection in far-field. The proposed metasurface achieves amplitude-phase modulation within a significantly wide bandwidth which covers 75–91.5 GHz and 99.3–115 GHz. By optimizing the coding patterns, the proposed graphene-based metasurfaces are able to not only realize 2-D beam steering, but also achieve beam switching from single beam to four beams at 87 GHz. The proposed design provides a novel solution for the flexible manipulation of millimeter waves, which can be applied to various fields such as vehicle radar, satellite communication, 6G wireless communication, and beyond.

## 1. Introduction

As the evolution of mobile communication technology has progressed from 1G to 5G, a multitude of wireless communication methods have emerged, encroaching upon scarce spectrum resources. In order to expand communication capacity, i.e., obtain wider bandwidth, future wireless networks will be compelled to venture into higher frequency bands, including millimeter wave and terahertz bands, which hold promise for the advancement of sixth-generation communication technologies (6G communication). Against this background, beam-reconfigurable antennas are increasingly being employed in personal communication devices, wireless local area networks, the Internet of Things, and 5G communication technologies. Traditional beam-reconfigurable antennas include phased array antennas [1], reflector antennas [2], and beam former antennas [3]. In recent years, with the rapid development of microstrip and metamaterials, beam reconfiguration can also be achieved through microstrip parasitic antennas [4] and metasurface antennas [5]. As a two-dimensional representation of metamaterials, metasurfaces, which are periodically arranged by artificial composite structures, have exceptional electromagnetic properties and have been widely adopted in the design of beam reconfigurable antennas.

Generally, depending on whether controllable elements or materials are added, the metasurface can be divided into active and passive types [6]. Once a passive metasurface is designed and fabricated, its electromagnetic characteristics cannot be changed. By loading active tunable elements or materials into the passive static metasurface unit, the electromagnetic beam can be dynamically modulated in real time. Active tunable components or materials include PIN diodes [7], varactor diodes [8], RF MEMS switches [9], liquid crystals [10], graphene [11], etc. PIN diodes and varactor diodes are diminutive in size and economical, thereby resolving the challenge of sluggish control velocity of electronic control machinery. This attribute is beneficial for devising multi-functional reconfigurable metasurface antennas with uninterrupted adjustment. Nevertheless, a significant impediment is that PIN diodes or varactor diodes demonstrate high losses and inadequate stability in high frequency applications. RF MEMS switches exhibit the merits of low power consumption, low insertion loss, high isolation, and excellent high frequency performance. However, they have the disadvantages of long switching time, high voltage drive, and high cost. The material of liquid crystal possesses low losses and anisotropic dielectric constants, which is an outstanding material for controllable media. Nonetheless, the extent of control of liquid crystal dielectric constants is narrow, thus restricting the overall regulation range of the system. Graphene exhibit a high degree of freedom in electrical modulation efficacy and also display singular and outstanding characteristics in optics, thermodynamics, and mechanics. Therefore, graphene is an excellent candidate for controllable metasurface in the high frequency band.

Initially, graphene has gained significant popularity in the optical and terahertz frequency bands owing to outstanding optical characteristics and the pliable and manageable surface conductivity over an extensive range of high frequencies. Chiang et al. demonstrated a broadband terahertz amplitude regulator based on complementary stacking of multilayer metamaterials, which shows a bandwidth of 0.5 THz centered at 1.25 THz, corresponding to a relative bandwidth of 40% [12]. A. Fallahi et al. discovered dynamically tunable Faraday rotation in the terahertz band using a graphene metasurface [13]. Lei Zhou et al. proposed and realized a new phase regulation metamaterial based on graphene–metal composite metamaterials [14], and achieved a maximum dynamic phase adjustment of 243° at 0.48 THz for the first time. Due to limitations in fabrication processes and other factors, application research pertaining to graphene in the microwave and millimeter wave range has been stagnant in its progression. Balci et al. used graphene capacitor structure to construct graphene flexible surfaces to achieve dynamic electric field control of microwave absorption, reflection and transmission at 10 GHz [15]. Leng T et al. fabricated a dipole antenna with graphene as the radiation subject on cardboard, which achieved the radiation target and had better flexibility than metal [16]. Based on graphene, H Chen et al. designed a reflective-phase-encoded metasurface operating at 6–14 GHz to achieve theta = ±60° beam steering and multibeam functionality [17]. Although some scholars have explored the application of graphene in the microwave band, the design of metasurfaces founded on graphene in the millimeter wave band is scarce.

In this work, we propose an active and reflective graphene-based metasurface in the W-band. To achieve beam reconfigurability, the reflection amplitude and phase of each metasurface unit is modulated by varying the Fermi level of graphene. And the coding bandwidth rages cover 75–91.5 GHz and 99.3–115 GHz. Under the premise that the two coding units have a certain reflection phase difference, beam steering and multi-beam switching can be achieved at a fixed frequency through the elaborate arrangement of the amplitude coding pattern. In comparison to conventional phased-array antennas, the graphene metasurface has the advantages of simple structure and light weight.

## 2. Theoretical Analysis

### 2.1. Graphene Conductivity

Graphene is a new type of high-performance material that was isolated from graphite in 2004 by Geim and Novoselov at the University of Manchester [18]. It is the first single-atomic layer 2D material to be prepared and processed in the laboratory. Graphene materials have a bipolar electric field effect [19]. Therefore, when an electric field is applied to the graphene material or chemical doping is performed, the carriers in the graphene crystal can be converted between electrons and holes [20]. As the voltage of the applied electric field can be seamlessly adjusted, the carrier concentration in the corresponding graphene can also be continuously regulated. This relationship can be expressed through Equation (1) [21,22].

(1) $$n={\left(n_{0}^{2}+\alpha^{2}|\Delta V{|}^{2}\right)}^{1/2}$$

where *n* is the carrier concentration in graphene, $n_{0}$ is the residual carrier concentration, and α is the capacitance of the control voltage gate, which is generally related to the electrode configuration. $|\Delta V|=\left|V_{CNP}-V_{g}\right|$, $V_{g}$ is the voltage of the applied electric field; $\left|V_{CNP}\right|$ is the compensation voltage when the concentration of carriers is 0.

The relationship between the Fermi level and carrier concentration of graphene at room temperature *T =* 300 K ($k_{B}T∼0.026\;eV$*)* is shown in Equation (2), where the Fermi velocity $v_{f}=10^{6\;}m\cdot s^{-1}$. If it belongs to the structure of the parallel plate capacitor, the concentration of carrier aggregation caused by the capacitance effect is $n=\epsilon_{0}\epsilon_{d}V_{g}/ed$.

(2) $$\left|E_{f}\right|=\hbar v_{f}(\pi n{)}^{1/2}$$

The relationship between relaxation time and Fermi level is shown in Equation (3). When other conditions have been determined, the relaxation time (τ) is proportional to the Fermi level $\left(E_{f}\right)$. When the Fermi level is higher, the relaxation time is longer. If the graphene carrier mobility $\mu =10^{4}{cm}^{2}V^{-1}s^{-1}$, the relaxation time can be calculated to be $0.1\times 10^{-12}$ s at the Fermi level $E_{f}=0$.1 eV.

(3) $$\tau =\mu E_{f}/ev_{f}^{2}$$

The effect of temperature on graphene carrier mobility is small, and the effect of impurity scattering on graphene carrier mobility is relatively large. Therefore, the graphene carrier mobility can be improved by increasing the purity of graphene at room temperature.

Kubo, a Japanese scientist, proposed that the Kubo formula [23] can be applied to modeling the transport characteristics of graphene. As shown in Equations (4)–(6), the conductivity of graphene is induced by its interband and intraband transitions. In the visible and near-infrared ranges, electrical tuning of the device is not possible, as graphene exhibits universal conductance (σ = $q^{2}$/4ℏ) dominated by interband transitions. However, from the mid-infrared to microwave bands, the conductivity of graphene can be dynamically controlled by electrostatic gating. Based on the Pauli Blocking effect [24], when the Fermi level *E_f_* > ℏω=2, the interband transition of carriers is forbidden, and intraband transitions have a significant influence on the dielectric properties of graphene layer. In this work, Graphene is used in the design of W-band metasurfaces, where its conductivity can be approximated by Equation (5).

(4) $$\sigma =\sigma_{\mathrm{intra}}+\sigma_{\mathrm{inter}}$$

(5) $$\sigma_{intra}=j\frac{q^{2}k_{B}T}{\pi \hbar \left(\hbar \omega +j\Gamma_{c}\right)}\left[\frac{E_{f}}{k_{B}T}+2ln\left(e^{-E_{f}/k_{B}T}+1\right)\right]$$

(6) $$\sigma_{\mathrm{inter}}=j\frac{q^{2}}{4\pi \hbar }ln\left[\frac{2\left|E_{f}\right|-\left(\hbar \omega +j\Gamma_{c}\right)}{2\left|E_{f}\right|+\left(\hbar \omega +j\Gamma_{c}\right)}\right]$$

where $E_{f}$ is the Fermi level, *q* is the fundamental charge, ω is the angular frequency, $K_{B}$ is the Boltzmann constant, *T* is the temperature, ℏ is the reduced Planck constant, τ is the relaxation time, and $\Gamma_{c}$ is the damping constant.

To summarize, the range of variation of graphene conductivity is limited by several factors, including Fermi level, carrier concentration, temperature, and operating frequency. Only by selecting the appropriate parameters can a large enough range of variation be achieved to ensure that the graphene surface switches between high and low impedance states. Based on Equation (5), the sheet impedance of graphene is inversely proportional to both carrier mobility and the Fermi level. Therefore, by applying a voltage to graphene, the concentration of carriers in graphene is controlled and the Fermi level of graphene will be changed. Eventually, the surface conductivity of graphene is manipulated. The adjustable Fermi level of graphene provides an idea for dynamic modulation of electromagnetic beams using graphene-based metasurfaces.

### 2.2. Graphene-Based Metasurface Coding

In 2014, Professor Cui proposed the notion of digital coding metasurface [25]. The metasurface units are binary processed using the numbers 0 and 1 to represent different amplitude or phase responses to electromagnetic waves. Generally, the coding metasurface is composed of N × M units with the same size. When a plane wave is incident vertically on a 1-bit reflective coded metasurface located in the Fresnel zone of the antenna [26], special far-field radiation direction maps can be formed by adjusting the phase and amplitude of the array units. The radius R of the Fresnel zone is described by Equation (7), where L represents the largest size of the antenna aperture, λ is the wavelength corresponding to the operating frequency.

(7) 0.62L3λ≤R≤2L2λ

Inspired by [27], the unit in the middle of the metasurface is taken as the origin and coordinates are defined as *mn*, −*mn*, and so on when expanding in different directions on both sides of the origin unit. The formula for far-field scattering is shown in Equation (8) according to the relevant conventional phased array antenna theory [28] and generalized Snell’s law [29].

(8) E(θ,φ)=f(θ,φ)∑x=0M2∑y=0N2Imnexp jk0dnsinθ+φ−mn+∑x=−M2−1∑y=−N2−1I−mnexp jk0dnsinθ+φ−mn

where θ and φ are the pitch and azimuth angles of the scattered field, f(θ,φ) is the far-field of the unit, $k_{0}$ is the propagation constant, and $I_{mn}\;and\;\varphi_{mn}$ are the amplitude and phase of the excitation current in the unit located at the coordinates (*x_m_*, *y_n_*), respectively. Similarly, $I_{-mn}$ and $\varphi_{-mn}$ are the amplitude and phase of the excitation current located at coordinates (−*x_m_*, −*y_n_*), respectively. The desired beam state is obtained when the coding units on the digital metasurface are arranged with reasonable planning.

## 3. Unit Design of Proposed Graphene-Based Metasurface

**Figure 1:**
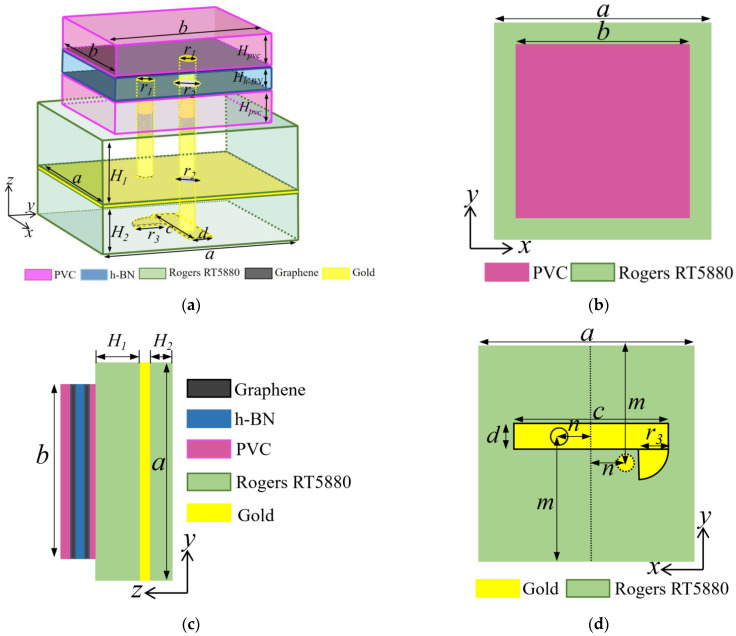
Metasurface unit structure. (**a**) 3D-view; (**b**) front view; (**c**) side view; (**d**) bottom view.

As shown in Figure 1, the unit of graphene-based metasurface consisted of two graphene layers, two substrate layers, two layers of polyvinyl chloride (PVC), a spacer and a ground layer. The dimensions of the graphene layer and the substrate were *a* and *b*, respectively. The relative permittivity for the PVC was 3.5 and the thickness was 0.07 mm. The dielectric substrate was Rogers RT5880 with a dielectric constant of 2.2 and a loss tangent of 0.0009. The thickness of the upper and the lower dielectric substrate was *H*_1_ and *H*_2_, respectively. To isolate two layers of graphene, a spacer was inserted between them. The spacer was made of hexagonal boron nitride (h-BN) with a thickness of 0.05 mm and the relative permittivity was 2.5. The DC feed was loaded between the upper and lower layers of the graphene. In this design, graphene was modeled and analyzed as a thickness-free 2-D material. The upper graphene served as the positive stage for DC excitation, while the lower graphene acted as the negative stage. Consequently, two vias with radius *r*_1_ were inserted into the substrate, as shown in Figure 1a. It should be emphasized that holes must be punched at the position where the longer via intersects with the lower graphene and the ground. The radius *r_2_* of the hole should be greater than the radius *r*_1_ of the via. Table 1 presents the unit parameters of the graphene metasurface. The practical application of DC feeding for each graphene unit was considered during the design process. The upper graphene layer has a via that connects to the back of the bottom dielectric substrate, enabling some electromagnetic waves to be transmitted to the bottom. In order to make the simulation results as close as possible to the actual application scenario, a DC feed-line was incorporated behind each unit and a fan structure was positioned next to the feeder as a low-resistance node that isolated the RF signal from the DC circuit.

**Figure 2:**
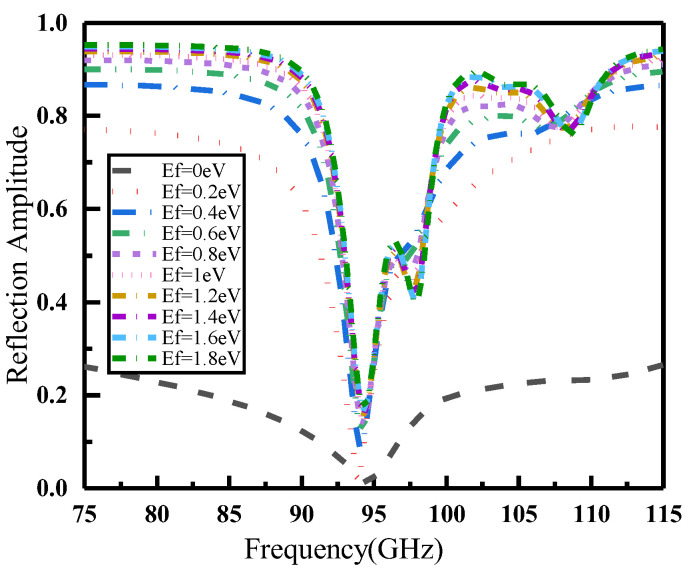
Reflection amplitudes of graphene metasurface unit at different Fermi levels.

To confirm the relationship between the phase and amplitude of the coding unit, the frequency domain simulator of the CST Studio Suite software was used to simulate and analyze the graphene metasurface unit. For this study, all discussions and results correspond to normal direction incidence scenario. The Fermi level of both upper and lower layers of graphene were simultaneously regulated, tuning it from 0 eV to 1.8 eV in steps of 0.2 eV. Figure 2 displays the reflection amplitudes corresponding to the graphene metasurface units at different Fermi energy levels. The unit reflection amplitude at the Fermi energy level of 0 eV was extremely low, but gradually increased with the applied Fermi level. This can be explained by Equation (5), as the conductivity of graphene was particularly low at the Fermi level of 0 eV, resulting in a significantly higher resistance value compared to that observed when the Fermi energy level state was applied. Therefore, in the absence of a Fermi level, the metasurface unit of graphene acted as an absorber, absorbing most of the electromagnetic waves and resulting in a low reflection amplitude of electromagnetic waves. When voltage was applied to the graphene, the resistance value of the metasurface unit gradually decreased, resulting in an increase in the reflection amplitude of electromagnetic waves. In amplitude encoding metasurfaces, the difference in amplitude between the two states should generally be greater than or equal to 0.5. Equations (1) and (2) show that the Fermi level of graphene is proportional to the applied electric field voltage. Figure 2 also demonstrates that the trend of increasing the reflection amplitude of the metasurface unit gradually slowed down as the Fermi level of graphene increases. Therefore, considering the coding standard and to avoid excessive applied voltages, we selected the graphene metasurface units with Fermi levels of 0 eV and 1 eV as encoding units “0” and “1”, respectively.

**Figure 3:**
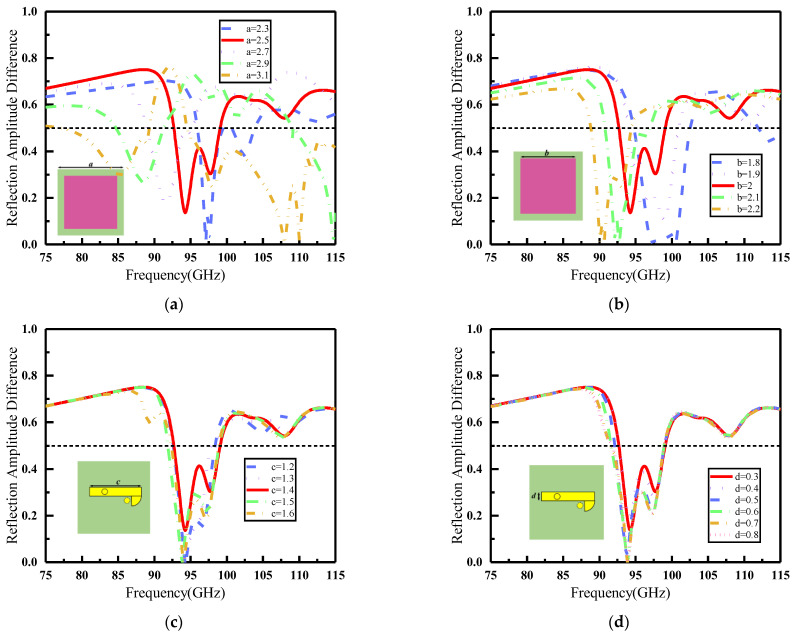
Influence of key parameters on the performance of metasurface coding unit. (**a**) Effect of substrate size a on unit performance when b = 2 mm, c = 1.4 mm, and d = 0.3 mm; (**b**) Effect of graphene size b on unit performance when a = 2.5 mm, c = 1.4 mm, and d = 0.3 mm; (**c**) Effect of bottom feed-line length c on unit performance when a = 2.5 mm, b = 2 mm, and d = 0.3 mm; (**d**) Effect of bottom feed-line width d on unit performance when a = 2.5 mm, b = 2 mm, and c = 1.4 mm.

Figure 3 depicts the impact of substrate size (a), graphene size (b), and bottom feed-line dimensions (c) and (d) on the encoding performance of metasurface unit reflection amplitude. The results in Figure 3a,b demonstrate that the parameters a and b have a significant impact on the band range of graphene metasurface reflection amplitude encoding. As a and b increase progressively, the frequency band satisfying the coding amplitude difference shifts towards lower frequencies, leading to a gradual reduction in the frequency band range. Additionally, Figure 3c,d indicate that while the size of the bottom feeder has a negligible effect on the amplitude difference between coding units, it is evident that c = 1.4 mm and d = 0.3 mm produce the highest amplitude difference between them. In conclusion, we determined that selecting key parameters such as a = 2.5 mm, b = 2 mm, c = 1.4 mm, and d = 0.3 mm yields the optimal encoding performance for metasurface unit reflection amplitude.

**Figure 4:**
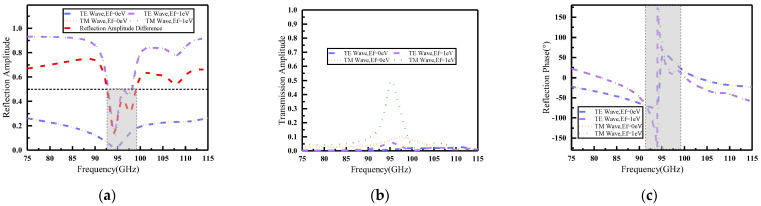
Comparison of amplitude and phase of different coding units under TE polarization wave and TM polarization wave irradiation. (**a**) Reflection amplitude; (**b**) transmission amplitude; (**c**) reflection phase.

Based on the data presented in Figure 4a,b, it can be observed that both the reflection amplitude and phase of the metasurface units remained constant regardless of whether they were excited by transverse electric (TE) or transverse magnetic (TM) polarized waves. This phenomenon can be attributed to the centrosymmetric structure of the metasurface unit, which rendered it insensitive to TE and TM polarization when illuminated normally by an incident electromagnetic wave. The graphical representation in Figure 4a clearly demonstrates that the amplitude of the coding unit “0” remained below 0.33 throughout the entire frequency range, while the amplitude of the coding unit “1” exceeded 0.8 for a significant portion of the frequency range. Additionally, it can be observed that the amplitude difference between the two states was generally equal to or greater than 0.5 across most bands within the frequency range of 75–115 GHz.

However, an exception can be seen within the shaded area of Figure 4a, which corresponds to frequencies between 92.5 and 99.3 GHz where the amplitude difference fell below 0.5, thus failing to meet the coding conditions. It should also be noted that during this particular frequency band, there was a gradual decline in the reflection amplitude of units, which ultimately approached 0. The results depicted in Figure 4b indicate that the transmission amplitude of both coding units was higher when subjected to incident TM polarization waves, particularly around 95 GHz. This phenomenon can be attributed to the placement of the bottom feeder along the x direction, which provided greater ease for the transmission of TM polarization waves while simultaneously suppressing TE polarization wave transmission. Additionally, when the metasurface was hit by a TM polarizing wave, the transmission amplitude of the unit 1 approached 0.5 around 95 GHz. The phenomenon reveals that half of the electromagnetic wave energy passed through the metasurface. This is one of the reasons that the reflection amplitude of the two units for electromagnetic waves decreased in the 92.5–99.3 GHz band. Secondly, Figure 4c shows that the phase of coding unit “1” and coding unit “0” mutated around 95 GHz. When the metasurface unit reflected an incident electromagnetic wave, the phase and amplitude changes interacted with each other, leading to the phenomenon that the amplitude decreased and then increased in the 92.5–99.3 GHz band for both types of coding units. In addition, due to the insertion of feeder posts and perforated structures, there was a certain reflection loss in the unit within that frequency band. As the frequency changed, the reflection pattern also changed, resulting in a sudden decrease in reflection amplitude.

In Figure 4c, the phase relationship between the two coding units is depicted, revealing a phase difference between the units in the non-shaded region (75–91.5 GHz and 99.3–115 GHz). In summary, the proposed graphene metasurface unit can be effectively coded for joint amplitude-phase modulation within the frequency bands of 75–91.5 GHz and 99.3–115 GHz.

## 4. Performance of Reconfigurable Graphene Metasurface

**Figure 5:**
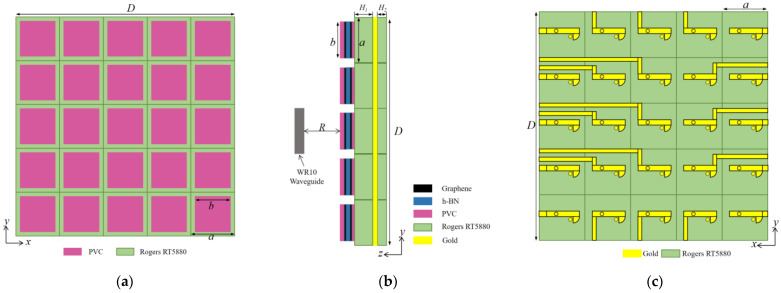
Graphene metasurface array. (**a**) Front view; (**b**) Side view; (**c**) Bottom view.

**Figure 6:**
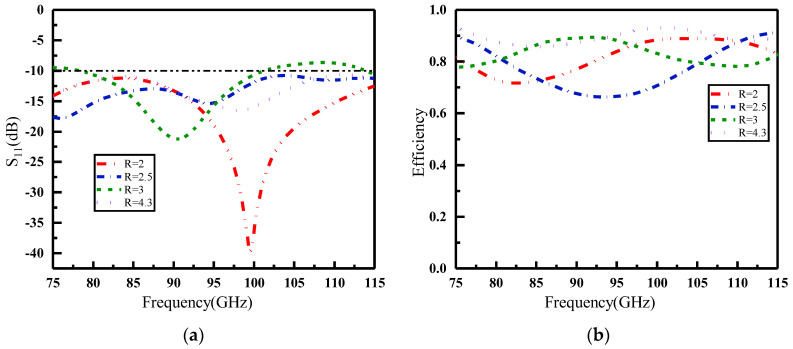
Performance with different distances between standard waveguide WR-10 and graphene metasurface of column code 11,000. (**a**) S11; (**b**) Efficiency.

As depicted in Figure 5, the graphene metasurface was fashioned into a 5 × 5 array by utilizing a total of 25 metasurface units. To verify its functionality, the conventional horn antenna was replaced with a wide-beam open-ended waveguide antenna (WR-10) due to its simple architecture and compact size. R is the distance between the standard waveguide WR-10 and the graphene metasurface. The metasurface designed in this paper operates in the W-band. According to Equation (7), *R* should be in the range of 1.56–4.96 mm. The selection of a reasonable focal length plays a crucial role in the overall metasurface performance. The time domain simulator of the CST Studio Suite software was used to simulate and analyze the graphene metasurface. Figure 6 shows the S11 and efficiency of the metasurface and waveguide at different distances in the state of column code 11,000. Although the performance improves with greater distance, at larger distances, the encoded metasurface becomes ineffective. It should be noted that the electromagnetic waves can be reflected as much as possible under the condition of low antenna thickness. Therefore, *R =* 2 mm is chosen as the final option. The metasurface dimension *D* = 12.5 mm, and the corresponding focal diameter ratio *R*/*D* = 0.16.

**Figure 7:**
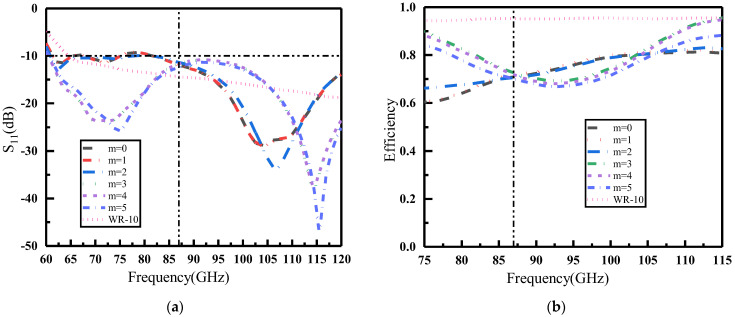
Performance comparison of antennas loaded with different states of metasurface. (**a**) S11; (**b**) Efficiency.

For convenience, we use “m” to denote the number of rows in the graphene metasurface unit “0” in the following simulation. The case where m = 0 corresponds to a situation that all rows are set to unit “1”; m = 1 means only row 1 is set to unit “0”; and m = 2 signifies that both rows 1 and 2 are in the “0” state and the rest in the “1” state, and so on. Figure 7 shows the S parameters and normalization efficiency of the graphene metasurface in different coding states. In the figure, the pink line depicts the S11 and normalization efficiency of the WR-10 without the loaded metasurface, while the remaining lines represent the WR-10 loaded metasurface in different coding states. In Figure 7a, it can be observed that the resonance point of the WR-10 shifted under different coding states after loading the graphene metasurface, though the bandwidth remained relatively constant. Furthermore, loading the graphene metasurface made the emission of electromagnetic waves in certain frequency bands more conductive. Loading the metasurface in different coding states changed the electrical characteristics of the feed antenna WR-10, which led to alterations in the interaction between the antenna and the metasurface. These alterations affected the magnitude of the S11 parameters and the frequency response. In Figure 7b, the normalization efficiency in most coding states is high. However, it was found that the efficiency decreased as the number of rows in which unit “0” is located increased. The main reason is the low conductivity of the unit “0” on the graphene metasurface and the low reflection amplitude of the electromagnetic waves. This phenomenon also leads to the low efficiency of the antenna when there are too many “0” units in the coded state.

To verify the reconfigurable performance of this graphene-based reflective encoded metasurface beam, we encoded the metasurface according to Equation (8). The operating frequencies led to different far-field functions of the subarrays, which in turn led to differences in the directionality functions of the entire hypersurface at different frequencies. Due to the fact that each unit of the metasurface in this design had two states, there were a total of 225 encoding and array methods for the entire array. Combining Figure 7a,b, the S11 and efficiency of different coding states at 87 GHz were more similar, so some coding states at 87 GHz frequency point were selected to demonstrate the metasurface function in this paper. The phase difference between the two coding units at 87 GHz could be inferred as △φ=30° according to Figure 4c. With the application of △φ and amplitude in Equation (6), the beam reconstruction can be obtained for different encoding cases. In the following 3D views, the square on the purple substrate is unit “1”, corresponding to graphene with a Fermi energy level of 1 eV; the square on the orange substrate is the unit “0”, which corresponds to graphene with a Fermi energy level of 0 eV.

**Figure 8:**
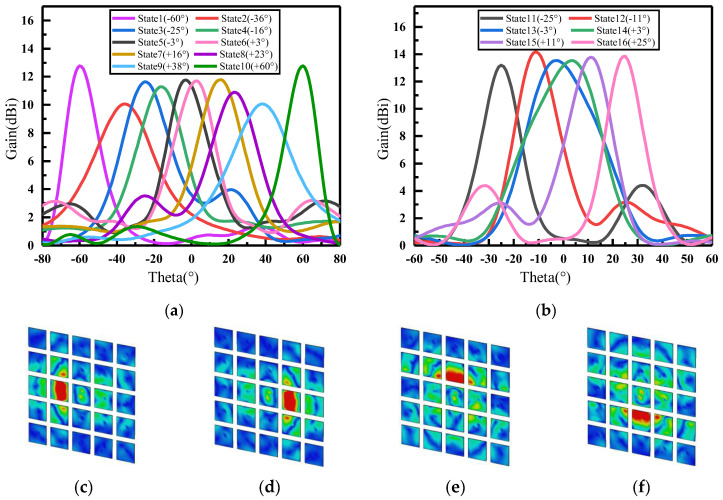
Single-beam steering at 87 GHz (**a**) in E-plane and (**b**) in H-plane; surface current of (**c**) state 3 (−25°); (**d**) state 8 (+23°); (**e**) state 11 (−25°); (**f**) state 16 (+25°).

As shown in Figure 8a,b, the effect of single-beam steering in the E-plane and H-plane of the antenna at 87 GHz is illustrated. By encoding in “columns”, the antenna was capable of achieving single-beam steering of ±60° in the E-plane, as demonstrated in Table 2. Similarly, by encoding in “rows”, single-beam steering of ±25° in the H-plane could be achieved, as shown in Table 3. The 0 and 1 in Table 2 and Table 3 correspond to the graphene units loaded at the Fermi energy level of 0 eV and 1 eV, respectively. Figure 8c–f illustrate the surface current distributions of the metasurface for states 3, 8, 11, and 16. It can be observed that the beam steering was directed towards the area where surface current was concentrated, which was caused by the conductivity difference between metasurface elements encoded with different amplitudes. When electromagnetic waves were incident on the metasurface, induced currents flowed from high-conductivity regions (i.e., unit 1) to low-conductivity regions (i.e., unit 0), and converged in areas of low conductivity.

**Figure 9:**
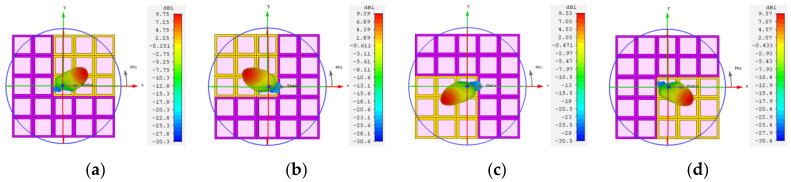
Three-dimensional radiation pattern of single-beam azimuthal rotation of 360° at 87 GHz. (**a**) (26°, 31°); (**b**) (26°, 148°); (**c**) (26°, 211°); (**d**) (26°, 330°).

**Figure 10:**
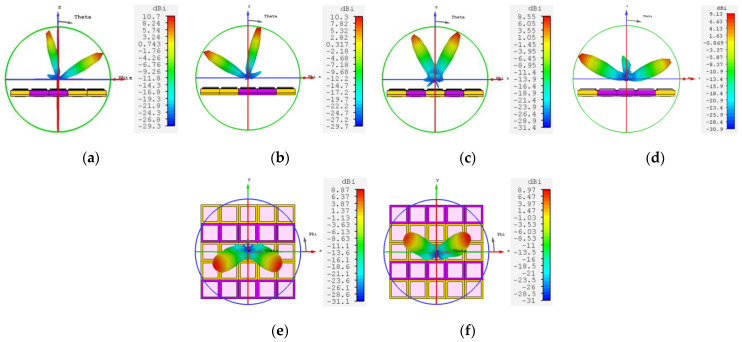
Three-dimensional radiation pattern of the dual-beam deflection at 87 GHz. (**a**) (−11°, 0°) and (60°, 0°); (**b**) (−60°, 0°) and (12°, 0°); (**c**) (−26°, 0°) and (27°, 0°); (**d**) (−64°, 0°) and (67°, 0°); (**e**) (43°, −25°) and (43°, −158°); (**f**) (−43°, −25°) and (−43°, −155°).

**Figure 11:**
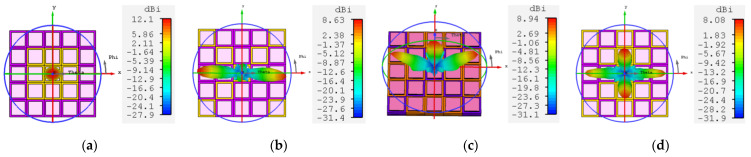

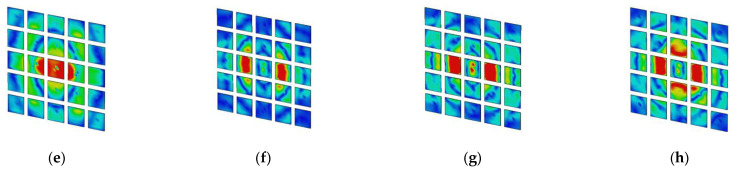
Three-dimensional radiation pattern of switching between one beam to four beams at 87 GHz (**a**) one beam; (**b**) two beams; (**c**) three beams; (**d**) four beams. Surface current of the corresponding coding state that (**e**) one beam; (**f**) two beams; (**g**) three beams; (**h**) four beams.

Figure 9 shows a single-beam azimuthal rotation of 360° in different encoding states, corresponding to beam steering coordinates of (26°, 31°), (26°, 148°), (26°, 211°), and (26°, 330°), respectively. In addition, dual-beam steering is possible at 87 GHz. Figure 10a–d show the dual-beam deflection realized in the E-plane, corresponding to dual-beam coordinates of (−11°, 0°) and (60°, 0°), (−60°, 0°) and (12°, 0°), (−26°, 0°) and (27°, 0°), (−64°, 0°) and (67°, 0°), respectively; Figure 10e,f show the dual-beam deflection realized in the D-plane, corresponding to the dual-beam coordinates of (43°, −25°) and (43°, −158°), (−43°, −25°) and (−43°, −155°), respectively. When the encoded state is no longer restricted to whole rows or columns, the metasurface is partitioned into multiple regions and the interaction between these regions produces multiple beams. Figure 11 illustrates the implementation of the metasurface for switching between one beam to four beams, along with the corresponding coding state surface current.

## 5. Performance Comparison

Table 4 shows the comparison between the metasurface proposed in this manuscript and other metasurfaces. The findings indicate that phase coding constitutes the bulk of the metasurface coding methods. Furthermore, the majority of metasurfaces that utilize graphene to accomplish beam refactoring operate in terahertz (THz). In this work, graphene was applied in millimeter-wave frequency bands to achieve the design of a broadband reflection amplitude-phase encoding metasurface. Additionally, the proposed metasurface has the capacity to execute beam steering and multi-beam switching concurrently.

## 6. Conclusions

In this work, a multifunctional metasurface with reflective amplitude-phase modulation is designed based on graphene. Graphene is commonly used in the terahertz band, and this work applies it to the W-band. The metasurface is composed of a 5 × 5 array consisting of only 25 units. By varying the Fermi level to encode the reflective amplitude and phase of the graphene metasurface, the metasurface has the capacity to execute beam steering and multi-beam switching concurrently. The graphene-based metasurface achieves beam reconstruction function over an exceptionally wideband from 75 to 91.5 GHz and 99.3 to 115 GHz. Compared to traditional phased array antennas, the graphene metasurface offers numerous advantages such as wide working bandwidth, simple structure, and lightweight design, making it an ideal candidate for vehicle-borne radar, satellite communication, and 6G wireless communication.

## Figures and Tables

**Table 1:** Graphene-based Metasurface Unit Parameters.

Parameter	a	b	c	d	*m*	*n*	*r*_1_	*r*_2_	*r*_3_	*H*_1_	*H*_2_	*H_pvc_*	*H_h-BN_*
Value (mm)	2.5	2	1.4	0.3	1.45	0.2	0.1	0.15	0.4	0.508	0.254	0.07	0.05

**Table 2:** The coded states mentioned in Figure 8a.

State	Column	#1	#2	#3	#4	#5
1	−60°	0	0	1	1	1
2	−36°	0	0	0	1	1
3	−25°	0	0	0	1	0
4	−16°	1	0	0	1	0
5	−3°	0	1	1	1	1
6	+3°	1	1	1	1	0
7	+16°	0	1	0	0	1
8	+23°	0	1	0	0	0
9	+38°	1	1	0	0	0
10	+60°	1	1	1	0	0

**Table 3:** The coded states mentioned in Figure 8b.

State	Row	#1	#2	#3	#4	#5
11	−25°	0	0	1	0	1
12	−11°	1	0	0	1	1
13	−3°	0	1	1	1	1
14	+3°	1	1	1	1	0
15	+11°	1	1	0	0	1
16	+25°	1	0	1	0	0

**Table 4:** Comparison of proposed work with other reported metasurfaces.

	[30]	[16]	[31]	[32]	[33]	This Work
Operating Frequency Band	2.57–2.64 GHz	6–14 GHz	0.56–0.74 THz, 0.75–0.98 THz and 0.99–1.08 THz	1.38–1.56 THz	1–1.4 THz	75–91.5 GHz, 99.3–115 GHz
Regulating components or materials	High dielectric fluid	Graphene	Graphene	Graphene	$\mathrm{Graphene}\;\mathrm{and}\;{VO}_{2}$	Graphene
Coding method	Phase	Phase	Amplitude and Phase	Amplitude	Phase	Amplitude and phase
Beam steering angle	Theta = ±20°	Theta = ±53.7°	Not mentioned	Six metasurfaces are arranged around the antenna to achieve phi = 360° beam steering	From theta = 0° to theta = 68.37°	Theta = ±60° in E-plane, theta = ±25° in H-plane, phi = 360° single-beam steering
Multi-beam status	Not mentioned	Four beams	Three beams	Three beams	Two beams	Four beams

## Data Availability

Data sharing is not applicable to this article.
